# Effects of Time Restricted Feeding and Meal Timing on An 8‐Week Aerobic Exercise Training Programme on Psychological Parameters—A Randomised Controlled Trial

**DOI:** 10.1002/ejsc.70017

**Published:** 2025-07-16

**Authors:** Florian Hofstätter, Martin Niedermeier, Linda K. Rausch, Justin S. Lawley, Martin Kopp

**Affiliations:** ^1^ Department of Sport Science University of Innsbruck Innsbruck Austria

**Keywords:** aerobic exercise, affect, intermittent fasting, quality of life

## Abstract

Exercise and short‐term fasting may have positive effects on psychological health. The aim of this study was to investigate effects of combining daily time restricted feeding and aerobic exercise on longer‐term psychological health parameters. Fifty‐two participants were randomised into three groups. (1) Training sessions were performed in the fasted state. (2) Training sessions were performed after consuming a standardised carbohydrate‐based snack. (3) Exercise training with an ad libitum diet as a control group. The 8‐week intervention consisted of daily time restricted feeding (16/8h) for both fasting groups and 3x/week à 60‐min aerobic training (∼73% of maximal heartrate) for all groups. Data were collected during pre‐ and post‐test using psychological questionnaires. A 3x2 mixed ANOVA with repeated measures was used to analyse pre to post differences. Thirty‐six participants were included in the analyses. No group‐by‐time interactions were found in any of the investigated parameters, suggesting that fasting groups showed similar effects as the control group. Significant main effects indicate increases in the *positive* subdomain of the Positive and Negative Affective Scale (*p* = 0.033; η^2^_p_0.130) and the subdomain *vital body dynamics* of the Body Image Questionnaire (*p* = 0.002; η^2^_p_ 0.022) over all groups. No significant main effects were observed in quality of life, eating behaviour, and mood. Data suggest that combining daily time restricted feeding and aerobic exercise similarly effects quality of life, mood, affect, as well as eating behaviour compared to exercise alone. Different combinations may be considered as a safe dietary option in conjunction with personal preferences.

## Highlights

- Daily time restricted feeding during exercise showed little to no group differences in affect, quality of life, and mood when compared to exercise alone and may not have any negative effects on chronic health and wellbeing in a young healthy population.
- Choosing fasted over fed exercise seems to be a safe option from a psychological point of view. Especially positive affect might be improved by combining time restricted feeding and exercise.
- Vital body dynamics (how a person perceives their own body) increased positively in all groups. Both fasting groups showed nonsignificant decreases in *eating concerns* scores. However, all other eating behaviour scores showed slight positive increases.

## 1 Introduction

Sport and exercise have positive effects on mental health, for example, depression, affect, or mood (Heissel et al. 2023; Ledochowski et al. 2017; Schuch et al. 2016). Current public health recommendations from the World Health Organisation suggest 150 min or more moderate aerobic exercise per week with both individual experimental interventions (Dunn et al. 2005) and large meta‐analyses of randomised controlled trials demonstrating a significant reduction in depressive symptoms (Kvam et al. 2016). Therefore, exercise should be offered as an evidence‐based treatment option for mental health (Heissel et al. 2023).

Additionally, fasting, the voluntary abstinence from food intake for a specified period of time is a known practice in religious and spiritual traditions, as well as one form of medical treatment that has become a well‐known form of lifestyle interventions (Fond et al. 2013; Sun et al. 2024). One example for a type of fasting gaining a lot of popularity is daily time restricted feeding (TRF), a diet strategy where daily calorie intake is restricted to a consistent feeding window (i.e., 8 h) and followed by a fasting window (i.e., 16 h) with no calorie intake, which can exert its beneficial effects by reducing body weight and fat mass through a reduction in total calorie intake, however calorie restriction is not necessarily the goal of TRF (Anton et al. 2018; Tinsley and La Bounty 2015). From a psychological point of view, studies have reported inconclusive results regarding effects of fasting. (Fond et al. 2013) reported in their review study that therapeutic fasting (e.g., up to 8 days and longer with less than 300 kcal/day) can lead to significant mood improvements, increased sleep quality and concentration. On the contrary, Wang and Wu (2022) stated that longer‐term fasting (> 2 days without calorie intake) might cause lower positive mood and decreased perceived work performance, probably which is probably caused by the constant distraction and not the act of fasting or the result of hunger. However, different to longer‐term fasting strategies, they pointed out that short‐term fasting, such as daily time restricted feeding (e.g., 16/8h intermittent fasting), has the potential to cause positive mood enhancement and might increase affective experiences, such as vitality, sense of achievement, reward or pride.

Physiological effects during fasted exercise have been investigated in several studies indicating that substrate utilisation such as fat oxidation during aerobic exercise or performance during resistance training can be positively influenced by the fed or fasted state (Hofstätter et al. 2025; Jeukendrup 2017; Vieira et al. 2016). However, it is unclear if psychological responses to exercise are similarly affected by the fasted or fed state. Previous studies have focused primarily only on acute psychological effects of single bouts of exercise on affective responses (e.g., gender/activity level differences in positive affect during exercise) but not on longer‐term or repeated training effects, or on effects of fasted versus fed exercise training (Aird et al. 2018; Kyral et al. 2019).

Both eating behaviour and physical activity have been reported to have reciprocal effects on quality of life, self‐esteem and overall mental health. For example, poor diets can negatively influence quality of life or self‐esteem, while the latter two both can lead to poorer individual diet quality (Bremner et al. 2020; De La Rie et al. 2005). Intermittent fasting and exercise have also been shown to have positive effects on body composition and body image (Tinsley and La Bounty 2015), as well as positive affect, which has been demonstrated to be a predictor for future engagement in physical activity (Bichler et al. 2022; Rhodes and Kates 2015; Williams et al. 2008). Positive affect during exercise might increase future engagement in physical activity and should therefore be an important aspect in health‐enhancing interventions (Kwan and Bryan 2010; Rhodes and Kates 2015; Williams et al. 2008). One might speculate that novel strategies to increase positive affect during physical activity might result in more engagement to future physical activity, and therefore lead to long term improvements in quality of life.

To the best of our knowledge, there are no studies in the literature investigating the effects of TRF combined with aerobic exercise over several weeks on psychological health parameters such as affect, quality of life, mood, or eating behaviour. Therefore, the aim of this study was to examine effects of an aerobic exercise programme combined with a daily time restricted feeding intervention on several psychological parameters (affect, quality of life, and mood). In addition, eating behaviour, subjective body image and self‐efficacy to physical activity will be investigated.

## 2 Methods

In the present study, we focus on psychological parameters mood, quality of life, eating behaviour, self‐efficacy to physical activity, body image, and mood in healthy young students.

Fifty‐two healthy, recreationally active students, who were registered in a study programme at the university at the time of this study (28 females) were recruited with an online questionnaire via the student university newsletter and personal contacts, whereby exclusion criteria such as participant's previous dietary habits were asked before enrolment in the study. Exclusion criteria were not being a registered student at our university, conducting a 16/8h (or similar) intermittent fasting regimen in the last 3 months prior to the study, pregnancy, being in lactation period, chronic or acute diseases, metabolic disorders and any history of mental disease. All participants read and signed written consent before starting the study during the pre‐test. The PAR‐Q was used to assess the physical activity readiness of study participants (Shephard et al. 1991). This project has been reviewed by the University Ethics Board and certified that it is in correspondence with all requirements of the ethical principles and the guidelines of good scientific practice stated in the Declaration of Helsinki.

The randomised controlled study consisted of three phases: a pre‐test phase, an intervention phase, and post‐test phase. The pre‐test included a selection of psychological questionnaires, anthropometric parameters (body height, weight, and body mass index), and an ergo‐spirometry to calculate each individual's exercise intensity that corresponded to their maximum fat oxidation (fat_max_‐test). This fat_max_‐test was conducted according to Achten et al. (2002), with 5 min stages á 20 W increases in workload until a respiratory exchange ratio of < 1.0 or subjective discomfort and determined the exercise intensity at which fat oxidation has the highest relative contribution to total energy expenditure. This method was chosen to provide the same relative workload for each individual that might not be influenced by a potential carbohydrate depletion due to intermittent fasting. Following the pre‐test, participants were randomised into three groups: 1) One group fasted for at least 14 h prior to training and exercised in the fasted state (fasting group, FG), 2) one group fasted for 16 h, but trained 30 min after consuming a small standardised carbohydrate‐rich snack (fasting but train fed group, FFG), 3) one exercise only control group, where participants could eat ad libitum (control group, CG). Regarding total daily fasting hours, the groups FG and FFG both followed the same 16/8‐h daily time restricted feeding protocol (16h of no calorie intake, 8h of eating and drinking ad libitum) and were advised not to change their regular diet in any other way than moving their usual meals into the pre‐defined feeding window. The FFG had to complete 16h of fasting before eating two provided standardised commercially available cereal bars 30 min before exercise (2 bars equal 50g, 212 kcal, fat 6.5 g, carb 33.5 g, fibre 1.9 g, protein 3.9 g, Salt 0.25 g). A high carbohydrate snack was chosen to counteract potential carbohydrate depletion from repeated TRF. For participant's convenience, FFG could take 2 bars with them for each upcoming training session. Fasting hours were documented with a diary filled in daily by all participants in the fasting groups. The CG was not given instructions what to eat prior to their exercise sessions and was asked not to mimic any diet similar to TRF. The study was completed with a post‐test 2 days after the last exercise session with the identical measurements and preconditions as in the pre‐test (time of day, overnight fast, no exercise for 24 h, no caffeine for 12 h).

After the pre‐test and randomisation, participants came into our gym on weekdays only to exercise on a direct‐drive weighted flywheel stationary bike (‘spinning bike’) 3x/week á 60 min for a total of 8 weeks under supervision of trained sport scientists. All participants trained at their individual fat_max_ heartrate zone (heartrate at 90%–100% of maximum rate of fat oxidation) (Achten et al. 2002). Heartrate during exercise was monitored with provided pulsoximeters (PM100, Medisana, part of Ogawa Smart Healthcare Technology Group Co. Ltd, China). All three groups performed the same exercise programme. The two fasting groups were allowed to move their fasting window by a few hours in accordance with their respective desired training hours for the following day (available training hours from 08.00a.m. to 01.00p.m. on weekdays resulting in a ‘breakfast‐skipping’ TRF schedule) but were advised to stick to a consistent schedule. FG had to complete at least 14h of fasting prior to exercise, for the participants to have enough time to comfortably finish exercise within the 16h fasting window before eating again (e.g., start exercise after 14 h of fasting, finish after 15 h, have one more hour to get home and then eat).

For the pre‐ and post‐test, a set of questionnaires described below was chosen to cover different aspects of psychological health each suited to assess certain domains of affect (main parameter), quality of life, well‐being, mood, body image, eating behaviour, and self‐efficacy towards physical activity (secondary parameters). Each questionnaire was explained specifically to give an answer that considers the past 4 weeks.

Our main parameter chronic affect was assessed using the Positive and Negative Affective Scale (PANAS) which consists of 10 positive and 10 negative associated feeling states (Watson et al. 1988). All items were rated on a five‐point Likert scale. Each 10 positive and negative items were then transformed into the respective subdomain ranging from one to five, where higher values indicate greater degree of affect. The PANAS has been studied as a valid, reliable and efficient questionnaire that can be used to assess both acute and chronic affective states (Cronbach's alpha = 0.88 (positive affect) and 0.87 (negative affect), PA‐NA intercorrelation = −0.17, test‐retest reliability 8 Weeks = 0.68 and 0.71 for PA and NA, respectively) (Watson et al. 1988).

Quality of life was assessed using the short version of the World Health Organisation's Quality of Life Questionnaire (WHOQOL‐BREF) consisting of 26 items covering 4 subdomains (physical health, psychological health, social relationships and environment) and 2 separate questions on overall quality of life and health satisfaction (Harper et al. 1998). All items were rated on a five‐point Likert scale. The 4 domain scores, as well as the 2 separate values were derived following the instructions from the manual and later transformed into a score of 0–100, where higher values indicated a higher QoL in the specific domain. The WHOQOL‐BREF was found to be a reliable, valid, and time‐efficient questionnaire for assessing overall quality of life (Cronbach's alphas = 0.87–0.68 for the 4 subdomains and *r* = 0.64–0.37, respectively) (Hawthorne et al. 2006).

The Eating Disorder Questionnaire (EDE‐Q8) is the modified eight‐item short form of the EDE‐Q (Fairburn and Beglin 1994) and is used to assess dietary behaviour such as restraint, eating concern, shape concern, and weight concern (Kliem et al. 2016). An example for the questions asked is ‘*Have you been deliberately trying to limit the amount of food you eat to change your body shape or weight*?’. All items are rated using a seven‐point Likert scale. Four subdomains and 1 total value each ranging 0–6 are derived by averaging the respective matching items, where higher values indicate higher degree of disturbed eating behaviour. The EDE‐Q8 correlates in its overall mean scores with those of the full 28‐item EDE‐Q (*r* = 0.97; Cronbach's alpha = 0.92 for men, 0.93 for women) (Kliem et al. 2016).

The questionnaire Self‐efficacy to Physical Activity is primarily suitable for research purposes in the field of health and sport psychology and assess a person's likelihood to engage in physical activity (Fuchs and Schwarzer 1994). It consists of 12 items rated using a 7‐point Likert scale. Each question asked consists of ‘*I'm confident to engage in a planned physical activity* …’ followed by 12 different given answers such as ‘… *when I'm feeling tired; … when the weather outside is bad*’. A total score is calculated by adding all values together, ranging 12–84, where a higher score indicates a higher degree of likelihood to engage in physical activity. The scale for recording self‐efficacy to physical activity has a high internal consistency (Cronbach's alpha = 0.89) and moderate correlation with comparable scales (*r* = 0.47) (Fuchs and Schwarzer 1994).

The Questionnaire on body image (FKB‐20) serves to record the cognitive‐affective evaluation of one's own body (Löwe and Clement 1996). It measures body image in two largely independent scales whose factor structure has been shown to be clearly replicable in different samples. It consists of 20 items rated using a 5‐point Likert scale. Sample items are ‘*Overall I feel robust and strong*’ or ‘*I am not satisfied with my body shape*’. Two subdomains (vital body dynamic and dismissive body image) are derived following the manual ranging 10–50. Higher scores indicate stronger expression in the respective domain. The questionnaire showed high internal consistency values for both subdomains (Cronbach's alpha = 0.84 and 0.91, respectively) (Albani et al. 2006).

The Mood Survey Scale (MSS) assesses eight categorical states (Activation, Elation, Contemplation, Calmness, Anger, Excitement, Depression, and Fatigue) (Abele‐Brehm and Brehm 1986). All items are rated using a five‐point Likert scale. The eight subscales were each calculated from five items, ranging 5–25. Higher values indicate stronger approval with each subscale. Internal consistency values for the subscales range between Cronbach's alpha = 0.70 and 0.88. Convergent validity coefficients for the subscales are between *r* = 0.54 to *r* = 0.87 (Abele‐Brehm and Brehm 1986).

A priori sample size calculation was performed using G*Power (3 × 2 mixed ANOVA with repeated measures; further assumptions: *α* = 0.05; 1‐β = 0.8; groups = 3). At the time of the study, we were not aware of any studies reporting effects of combining time restricted feeding and aerobic exercise during a multi‐week intervention on acute or chronic affect or well‐being. Therefore, a moderate effect size was taken as default. However, the sample size in the present study is in a similar range of previously published studies on effects of exercise in the fasted versus fed state (Vieira et al. 2016). It seemed realistic to assume a drop‐out rate of 20%, setting the final sample size at *n* = 52.

Pre to post differences were analysed with Jamovi v2.3 using a series of 3 × 2 mixed ANOVAs (The Jamovi Project 2022). The between‐subject factor group contained 3 levels: CG, FG, FFG. The within‐subject factor time contained 2 levels: pre‐test, post‐test. Level of significance was set to *p* < 0.05. Bonferroni corrections were used to account for increased possibility of type‐I error. Although data was not normally distributed, ANOVA is considered a robust test procedure (Blanca et al. 2017). Results were double‐checked using Kruskal‐Wallis‐Tests as a nonparametric test, supporting ANOVA results.

## 3 Results

Inclusion criteria during data analysis were a compliance rate of 80% or more for exercise sessions for all groups, and 80% of completed fasting days for the fasting groups (Table 1). Out of 52 participants, 15 dropped out during the intervention phase, (12 participants because of time problems, two because of personal reasons, one because of health issues), and one participant with exercise compliance of under 60% was excluded during data analyses. This excluded participant was later added to the total number of drop‐outs (drop‐outs *n* = 15; exclusions *n* = 1) leading to a final drop‐out rate of 30.7% (*n* = 16; FG *n* = 6, FFG *n* = 5, CG *n* = 5). In the end 36 participants (28 females, 8 males; age 24.4 ± 3.9 years; height 168.6 ± 6.4 cm; weight 65.7 ± 10.0 kg; body mass index 23.0 ± 2.6 kg/m^2^) were included in the analysis. Full participants characteristics, compliance rates, number of training sessions, and fat_max_ heartrate are presented in Tables 1 and 2.

**TABLE 1:** Study compliance, number of training sessions, and heart rate for each group after apply exclusion criteria.

					Effect sizes		
	CG (*n* = 10)	FFG (*n* = 12)	FG (*n* = 14)	*p*‐value	CG ‐ FFG	CG ‐ FG	FFG ‐ FG
Fasting compliance (%)	n.a.	89.4 ± 6.2	92.1 ± 6.0	0.279	n.a.	n.a.	0.436
Exercise compliance (%)	88.3 ± 7.6	87.5 ± 5.3	85.1 ± 5.6	0.397	0.139	0.534	0.395
Total training sessions (n)	21.2 ± 1.8	21.0 ± 1.2	20.4 ± 1.3	0.397	0.138	0.533	0.395
Fat_max_‐HR	142.8 ± 10.8	140.3 ± 15.2	146.2 ± 12.6	0.519	0.193	0.259	0.452

*Note:* Data presented as mean ± standard deviations. *p*‐values represent ANOVA results; effect sizes are presented as Cohen's d.

Abbreviations: CG, control group; FFG, fasted fed group; FG, fasted group.

**TABLE 2:** Participant characteristics for each group after applying exclusion criteria.

					Effect sizes		
	CG (*n* = 10)	FFG (*n* = 12)	FG (*n* = 14)	*p*‐value	CG ‐ FFG	CG ‐ FG	FFG ‐ FG
Sex (m/f)	4/6	3/9	1/13	—	—	—	—
Age (years)	24.8 ± 5.1	24.2 ± 3.9	24.2 ± 3.4	0.922	0.156	0.145	0.012
Height (cm)	167.9 ± 5.7	171.2 ± 8.1	166.9 ± 4.7	0.217	0.529	0.154	0.683
Weight (kg)	68.2 ± 8.9	67.7 ± 13.9	62.1 ± 5.3	0.239	0.050	0.618	0.568
BMI (kg/m^2^)	24.2 ± 3.3	22.9 ± 2.7	22.3 ± 1.8	0.203	0.522	0.751	0.229

*Note:* Data presented as mean ± standard deviations. *p*‐values represent ANOVA results; effect sizes are presented as Cohen's d.

Abbreviations: CG, control group; FFG, fasted fed group; FG, fasted group.

**FIGURE 1:**
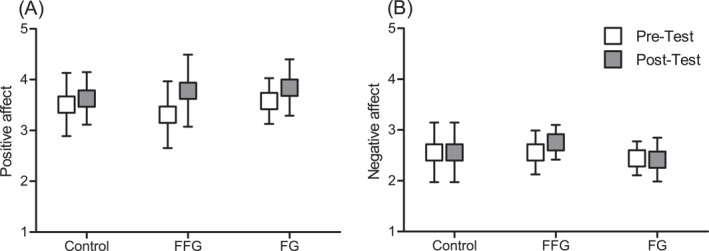
Comparison of positive (A) and negative (B) affect from pre‐to post‐test split by groups; error bars represent standard deviation.

A significant main effect in the PANAS subdomain positive affect was observed (*p* = 0.033, partial *η* ^2^ = 0.130), indicating an increase in positive affect (Figure 1a). No significant main effect was found in the subdomain negative affect (*p* = 0.669; partial *η* ^2^ = 0.006) (Figure 1b). No significant group‐by‐time interactions were found between pre‐ and post‐test in both subdomains of the PANAS (positive affect *p* = 0.554, partial *η* ^2^ = 0.035; negative affect *p* = 0.249, partial *η* ^2^ = 0.081). However, post hoc sample size analyses suggest a potential significant group‐by‐time difference in the positive domain with a total sample size of *n* = 72 (*f* = 0.190; *α* = 0.05; 1‐β = 0.8).

No significant main effects or group‐by‐time interactions were found in WHO‐QOL‐BREF subdomains health, quality of life, physiological health, psychological health, social relationships and environment (Table 3).

**TABLE 3:** Change scores (Δ) from pre‐to post‐test of secondary parameters.

	Control	FFG	FG	RM	Partial	RM*group	Partial	Between subject	Partial
	Δ	Δ	Δ	*p*‐value	*η* ^2^	*p*‐value	*η* ^2^	*p*‐value	*η* ^2^
Quality of life questionnaire									
Overall health	7.5 ± 16.9	8.3 ± 19.5	0.0 ± 13.9	0.070	0.032	0.388	0.018	0.630	0.028
Quality of life	7.5 ± 12.1	0.0 ± 18.5	3.6 ± 21.6	0.241	0.014	0.638	0.009	0.989	0.001
Physiological health	1.4 ± 6.8	3.3 ± 10.4	1.0 ± 7.6	0.188	0.010	0.780	0.003	0.844	0.010
Psychological health	3.3 ± 6.5	3.5 ± 8.5	2.4 ± 10.7	0.050	0.017	0.945	0.000	0.957	0.003
Social relationships	2.5 ± 11.2	0.7 ± 6.6	−3.6 ± 17.2	0.954	0.000	0.492	0.008	0.459	0.046
Environment	−3.4 ± 5.2	−0.3 ± 4.1	−2.5 ± 9.6	0.091	0.015	0.550	0.006	0.399	0.054
EDE‐Q8									
Restraint	−0.5 ± 1.0	−0.4 ± 1.0	−0.4 ± 1.5	0.051	0.016	0.994	0.000	0.742	0.018
Eating concern	−0.3 ± 0.6	0.1 ± 0.8	0.4 ± 1.2	0.570	0.002	0.259	0.015	0.267	0.077
Shape concern	−0.3 ± 1.6	−0.5 ± 0.9	−0.2 ± 1.0	0.102	0.012	0.778	0.002	0.463	0.046
Weight concern	−0.2 ± 0.9	−0.6 ± 1.2	−0.1 ± 1.3	0.183	0.007	0.513	0.005	0.393	0.055
Total	−0.3 ± 0.6	−0.3 ± 0.7	−0.1 ± 0.7	0.037	0.010	0.555	0.003	0.611	0.029
FKB‐20									
Dismissive attitude	0.2 ± 2.0	0 ± 5.1	−1.5 ± 5.0	0.565	0.001	0.578	0.002	0.774	0.015
Vital body dynamics	1.7 ± 2.1	2.3 ± 4.2	2.1 ± 4.0	0.002*	0.022	0.923	0.000	0.437	0.049
Self‐efficacy towards physical activity									
Total	−0.7 ± 5.1	3.2 ± 13.9	−5 ± 9.3	0.628	0.002	0.144	0.031	0.166	0.103
Mood survey scale									
Activity	−1.4 ± 4.1	−0.3 ± 2.3	−0.5 ± 4.1	0.244	0.008	0.740	0.003	0.775	0.015
Elation	−0.3 ± 4.7	−0.2 ± 2.1	−1.4 ± 5.6	0.405	0.005	0.733	0.005	0.586	0.032
Contemplation	0.6 ± 2.6	1.1 ± 2.7	1.1 ± 3.1	0.065	0.022	0.905	0.001	0.856	0.009
Calmness	−0.6 ± 2.8	0.9 ± 3.4	−2 ± 3.8	0.335	0.005	0.109	0.026	0.595	0.031
Anger	0.8 ± 2.9	−1.3 ± 2.1	2.1 ± 3.9	0.312	0.008	0.036	0.057	0.488	0.043
Excitement	0.4 ± 1.8	−1 ± 2.6	−0.3 ± 3.6	0.542	0.003	0.522	0.009	0.836	0.011
Depression	0.8 ± 4.1	−1.2 ± 1.9	1.4 ± 3.5	0.549	0.002	0.140	0.028	0.133	0.115
Fatigue	1.4 ± 4.3	0.5 ± 3.2	1.9 ± 2.6	0.032	0.024	0.582	0.005	0.517	0.039

*Note:* Data presented as mean ± standard deviation. repeated measures (RM), Eating Disorder Questionnaire 8‐Items short‐from (EDE‐Q8), Body Image Questionnaire (FKB‐20) are presented as means ± standard deviation.

* Significance.

After applying Bonferroni corrections, levels of significance were adapted to *p* < 0.008 for FKB‐20, *p* < 0.013 for EDE‐Q8, and *p* < 0.006 for MSS, respectively. Therefore, no secondary parameters showed significant main effects or group‐by‐time interactions, except FKB‐20's subdomain vital body dynamics, which showed a significant main effect (*p* = 0.002, partial *η* ^2^ = 0.022), indicating improvements in this subdomain in all three groups (Table 3).

## 4 Discussion

Manipulating diet and/or incorporating aerobic exercise are modifiable lifestyle interventions that may serve as a strategy to improve or maintain mental health in a broader population. The main focus of the study was to provide new insights on the effects of daily time restricted feeding in addition to moderate aerobic exercise on different psychological health parameters, as no studies in the literature investigated these combined effects to this date. Overall, the data suggest that combining TRF and exercise has similar effects on psychological parameters in a healthy population compared to exercise alone.

Previous studies often demonstrated positive mental effects from fasting or exercise in populations with mild to moderate major depressive disorders or mood disturbances. For example, Fond et al. (2013) reported that fasting might lead to improved subjective well‐being and increased mood, while Hussin et al. (2013) reported decreases in tension, anger and total mood disturbances through fasting with calorie restriction, but no differences in depression scores. Additionally, Kvam et al. (2016) demonstrated large positive effects on depression scores after exercise. However, our study population showed relatively high scores in most of the investigated domains from the beginning. Therefore, any lack of further improvements might have been due to the healthy study population. Our data suggest no differences between fasting groups regarding psychological responses compared to previous physiological studies, which have shown that acute metabolic responses occur when fed versus fasted exercise was compared (Vieira et al. 2016). However, a systematic review found, that food timing can affect circadian rhythm and therefore might influences cognitive performance, mood regulation or sleep quality (Réda et al. 2020). Although our study compared two different combinations of TRF and exercise, the actual magnitude of the difference in food timing in this study might not be enough, to show any effects regarding chrono nutrition.

The scores in the positive subdomain of the PANAS were comparable to healthy populations in the literature in the beginning of the study and still showed positive improvements over the course of the intervention, though no significant group differences were observed, indicating that fasting had no significant impact on the improvement in positive affect (Watson et al. 1988). WHO‐QoL scores were all comparable to healthy people in the literature, however, the authors want to point out that after our intervention FG showed a slight negative decrease and CG a slight positive increase in the subdomain social relationships, even though no significant differences were shown (Skevington et al. 2004). We think this is worth mentioning, because verbal feedback from participants during the course of the study indicated concerns about both restrictions in social relationships because of their respective fasting hours and pandemic related restrictions in the early phase of the study. Slightly higher scores in the negative subdomain of the PANAS in the beginning of the study (∼2.5 vs. 1.82) compared to the literature might also be related to pandemic restrictions, although no significant changes over the course of the intervention were shown (Watson et al. 1988).

No significant changes in the mood survey scale over the course of the study support the literature, that mood might be affected acutely by different internal or external factors but does not seem to be chronically affected by the combination of the two types of interventions in the underlying study (Abele‐Brehm and Brehm 1986).

Self‐efficacy towards physical activity levels were higher compared to the literature (total scores of 4.87–5.68 vs. 3.92), suggesting some potential recruitment bias in the form of pre‐existing interest in the topics intermittent fasting and/or aerobic exercise (Fuchs and Schwarzer 1994). The overall compliance rates of 85% for exercise participation and over 90% for fasting days support this assumption. Significant main effects in vital body dynamics in the FKB‐20 suggest positive improvements in how individuals perceive their own bodies. However, no significant group‐by‐time differences indicate that fasting had no impact on these improvements. According to Löwe and Clement (1996) body image is difficult to assess verbally, but their study has validated that the FKB‐20 shows the clinically relevant aspects of body image. We therefore suggest that exercise had a valid positive impact on participant's body image regardless of the fed or fasted state, as has been demonstrated in previous studies (Zaccagni and Gualdi‐Russo 2023).

The subdomains in the EDE‐Q8 were slightly higher in the beginning when compared to the literature, indicating potential pre‐existing attention to eating behaviour in all participants but all showed slight positive nonsignificant developments, except the subdomain eating concern which showed slight nonsignificant negative developments for both fasting groups, respectively (Hilbert et al. 2007). It remains unclear if this effect is solely due to the time constraints of the intervention or an increased overall attention to eating and drinking.

## 5 Limitations

The authors are aware that the study took place in a time when pandemic restrictions were still upheld but lifted over the course of the study. Therefore, any main effects might have been influenced by external, for example, end of pandemic related social restrictions, and nonstudy related factors, that could not have been accounted for during data collection.

Although we had no ‘fasting but no exercise group’ or a ‘no intervention control group’, null findings in most domains do not suggest a need for additional control groups. Still, we would like to point out, that in different study populations, for example, mentally or physically unhealthy people, where greater effects might be expected, an additional control group could provide clearer insights in the effects of the intervention.

For participants' convenience continuous glucose monitors were not used for this study, therefore, fasting hours were only monitored handwritten. For future studies a documentation less prone to potential errors or inaccuracies is recommended. Additionally, we did not collect data on total calories consumed per day, and we did not assess macronutrients in participant's diets. Therefore, one limitation between the groups were potential calorie or carbohydrate restrictions in their respective diet. Secondly, due to the timing of exercise and fasting for FFG, this group only had 6.5 h for proper eating on training days, as they started their eating window with a relatively low caloric snack, before waiting 30 min until training for an additional 60 min. This might have caused a greater potential calorie restriction in the FFG group. Slightly greater loss in body weight (FFG, −1.9 ± 2.29 kg; FG, 0.46 ± 1.03 kg; CG, −0.64 ± 0.75 kg) in the FFG group would support this idea, that total calorie intake was lower, but as we did not monitor calorie intake or dietary habits, we do not want to speculate if this effect was caused by the shorter eating window for FFG or by other potential dietary changes in all groups.

## 6 Conclusion

Our data suggest that combining TRF and exercise training shows similar effects on psychological parameters when compared to exercise alone. It seems that meal timing and/or time of exercise does not ameliorate potential positive mental effects. In healthy young students this combination might provide a safe and individually preferred modality for an active and healthy lifestyle. We propose that healthy young individuals may safely integrate daily intermittent fasting regimens (e.g.,: 16/8 fasting) into their everyday life and combine it with moderate aerobic exercise in line with personal timing preferences. For potential health benefits in unhealthy or at‐risk populations the authors suggest further research in people with lower scores in quality of life, or with certain health risks such as obesity, eating disorders or mental health problems to see if potential positive effects might still occur.

## Author Contributions

**Florian Hofstätter:** conceptualization, methodology, formal analysis, investigation, writing – original draft, visualization, project administration, funding acquisition. **Martin Niedermeier:** formal analysis, writing – review and Editing. **Linda K. Rausch:** writing – review and Editing. **Justin S. Lawley:** writing – review and editing, supervision. **Martin Kopp:** writing – review and editing, supervision.

## Ethics Statement

This study was reviewed by the University Ethics Board at Innsbruck University (#91/2021) and conformed to the ethical principles and guidelines of good scientific practice stated in the Declaration of Helsinki, except for registration in a database. All participants provided written informed consent before enrolment in the study.

## Conflicts of Interest

The authors declare no conflicts of interest.

## Data Availability

The data that support the findings of this study are available from the corresponding author upon reasonable request.
